# Finding the Needle in the Haystack—the Use of Microfluidic Droplet Technology to Identify Vitamin-Secreting Lactic Acid Bacteria

**DOI:** 10.1128/mBio.00526-17

**Published:** 2017-05-30

**Authors:** Jun Chen, Mike Vestergaard, Thomas Glasdam Jensen, Jing Shen, Martin Dufva, Christian Solem, Peter Ruhdal Jensen

**Affiliations:** a National Food Institute, Technical University of Denmark, Lyngby, Denmark; b Department of Micro- and Nanotechnology, Technical University of Denmark, Lyngby, Denmark; Korea Advanced Institute of Science and Technology

**Keywords:** classical mutagenesis, droplet microfluidics, lactic acid bacteria, high-throughput screening, vitamin B_2_

## Abstract

Efficient screening technologies aim to reduce both the time and the cost required for identifying rare mutants possessing a phenotype of interest in a mutagenized population. In this study, we combined a mild mutagenesis strategy with high-throughput screening based on microfluidic droplet technology to identify *Lactococcus lactis* variants secreting vitamin B_2_ (riboflavin). Initially, we used a roseoflavin-resistant mutant of *L. lactis* strain MG1363, JC017, which secreted low levels of riboflavin. By using fluorescence-activated droplet sorting, several mutants that secreted riboflavin more efficiently than JC017 were readily isolated from the mutagenesis library. The screening was highly efficient, and candidates with as few as 1.6 mutations per million base pairs (Mbp) were isolated. The genetic characterization revealed that riboflavin production was triggered by mutations inhibiting purine biosynthesis, which is surprising since the purine nucleotide GTP is a riboflavin precursor. Purine starvation in the mutants induced overexpression of the riboflavin biosynthesis cluster *ribABGH*. When the purine starvation was relieved by purine supplementation in the growth medium, the outcome was an immediate downregulation of the riboflavin biosynthesis cluster and a reduction in riboflavin production. Finally, by applying the new isolates in milk fermentation, the riboflavin content of milk (0.99 mg/liter) was improved to 2.81 mg/liter, compared with 0.66 mg/liter and 1.51 mg/liter by using the wild-type strain and the original roseoflavin-resistant mutant JC017, respectively. The results obtained demonstrate how powerful classical mutagenesis can be when combined with droplet-based microfluidic screening technology for obtaining microorganisms with useful attributes.

## IMPORTANCE

The food industry prefers to use classical approaches, e.g., random mutagenesis followed by screening, to improve microorganisms used in food production, as the use of recombinant DNA technologies is still not widely accepted. Although modern automated screening platforms are widely accessible, screening remains as a bottleneck in strain development, especially when a mild mutagenesis approach is applied to reduce the chance of accumulating unintended mutations, which may cause unwanted phenotypic changes. Here, we incorporate a droplet-based high-throughput screening method into the strain development process and readily capture *L. lactis* variants with more efficient vitamin secretion from low-error-rate mutagenesis libraries. This study shows that useful mutants showing strong phenotypes but without extensive mutations can be identified with efficient screening technologies. It is therefore possible to avoid accumulating detrimental mutations while enriching beneficial ones through iterative mutagenesis screening. Due to the low mutation rates, the genetic determinants are also readily identified.

## INTRODUCTION

Random mutagenesis is a nondirected and powerful approach for obtaining useful variants of living organisms, including the less well-characterized ones. Random mutagenesis is also valuable when the objective is to obtain cell factories with superior performance, although research over the past two decades has rapidly expanded the knowledge about metabolism and the development of genetic tools, enabling more targeted optimization of cellular metabolism (1). Cellular metabolism is highly complex, and it is difficult to predict the consequences of a particular perturbation or which methods to use for perturbing metabolism to achieve a desired outcome. Therefore, approaches involving both random mutagenesis and targeted metabolic engineering are often used for solving different optimization challenges, particularly within the biotechnology industry (1). The harshness of the mutagenesis conditions dictates the mutation frequency, and the objective is often to achieve the highest possible mutation frequency in order to facilitate screening for mutants with the desired traits. A trade-off is the introduction of numerous other mutations, some of which might affect overall fitness (2). When using a milder form of mutagenesis, the reduced mutation frequency necessitates a more efficient screening approach. As an illustration of this problem, tens of thousands of conventional 96-well microplates are required to select a mutant bearing a specific point mutation from a UV-mutagenized *Escherichia coli* library, where the mutation frequency is 10^−6^ (3). The screening volume has been reduced to 1 µl using higher-throughput microplates with 1,536 wells, but evaporation and the capillary effect often become prohibitive factors (4). In practice, robotics is also required to be able to perform high-throughput screening (HTS) using microplates, but a generally low operating rate is a major limitation (5).

Droplet-based microfluidics (DBM) has become an attractive alternative to plates and robotics for HTS, as the reaction mixture volumes are very small and droplets are sorted at high speed (6). Generally, cells are captured in a surfactant-stabilized aqueous sphere surrounded by an immiscible oil phase. In contrast to bulk-based fluorescence-activated cell sorting (FACS), cells freely proliferate and accumulate extracellular products in an isolated compartment, and the droplets are individually screened for their contents using different approaches (7). The small size of the droplets, whose volumes are measured in nanoliters or picoliters, allows affordable HTS with much reduced costs for consumables (8). DBM has been used in several screening applications, such as optimization of cell factories for the production of both recombinant proteins and chemicals (6, 7, 9–11). Some studies have also applied this technique to screen libraries generated using whole-genome random mutagenesis (10, 11). For example, Huang et al. (10) used droplet sorting to identify yeast mutants from a UV-mutagenized library that more efficiently secreted amylase. Their genome resequencing results reveal that a total of 330 mutations (approximately 30 mutations/million base pairs [Mbp]) had accumulated in the mutant, where only a few mutations have been found tightly correlated to the observed changes in protein secretion. It is also possible to use a mild mutagenesis strategy that introduces a few variations in the chromosome, which is a more demanding application requiring a higher-throughput screening. Thus, the question of whether the microfluidic screening approach is suitable for this challenge has not yet been completely addressed.

On the other hand, metabolic engineering is often not an option for improving the microorganisms used within food-related industries, e.g., the dairy and brewing industries, due to regulations and consumer resistance that prevent the use of genetically modified organisms (GMO) in food products (12). In this case, traditional whole-genome random mutagenesis followed by screening and selection is still the preferred method for optimizing performance. A good example is lactic acid bacteria (LAB), which are used extensively within the dairy industry for the manufacture of fermented milk products (5).

In milk fermentation, LAB produce lactic acid and other aroma compounds contributing to taste and flavor formation in the finished products. In addition, they are able to change the levels of certain micronutrients in fermented milk through relevant metabolic activities (13). An example is riboflavin, a B group vitamin, which is used both as a supplement for food and as a natural food color (E-101) (13). This essential vitamin is ingested by humans through their daily diets, but deficiency (ariboflavinosis) is still a common phenomenon in both developed and developing countries and causes several symptoms, such as hyperemia and cheilosis (13). The use of chemical selection to obtain non-GMO riboflavin overproducers from LAB, e.g., for use in the fortification of fermented food, has been a popular approach that has been used for many years, but further improvement is limited by a lack of efficient screening tools (13, 14). Here, we overcome this limitation by implementing DBM to screen a mildly mutagenized library of the lactic acid bacterium *Lactococcus lactis* for candidates with improved riboflavin production.

## RESULTS

### Generation of a riboflavin overproducer using roseoflavin selection.

*L. lactis* normally does not accumulate riboflavin extracellularly due to the strict regulation of its biosynthesis, which is mediated by a flavin mononucleotide (FMN) riboswitch. However, deregulated mutants are easily isolated by selecting for resistance toward the riboflavin analogue roseoflavin (14). We used this approach and successfully isolated the mutant strain JC017, which produced small amounts of riboflavin (0.82 mg/liter) when grown in synthetic medium containing 0.5% glucose. The DNA sequencing results indicated that the mutant lacked a small chromosomal region (reference position, 1504499 to 1504614) harboring the FMN riboswitch that precedes the riboflavin biosynthesis cluster (15).

### Random mutagenesis of the roseoflavin-resistant mutant and microfluidic screening.

**FIG 1:**
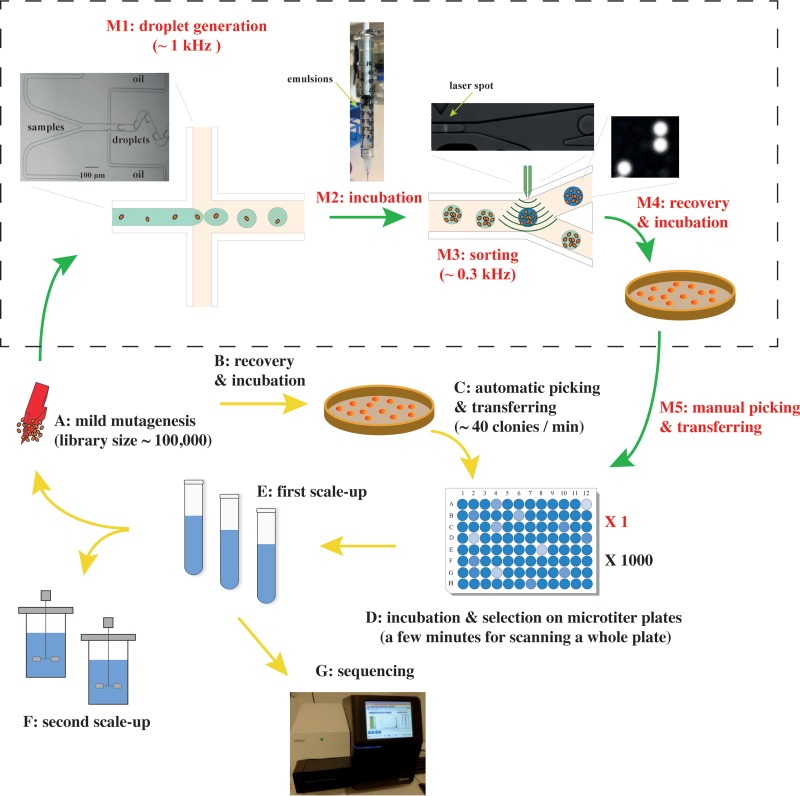
Incorporation of the droplet-based microfluidic screening toolbox into the classical strain development process based on random mutagenesis and microplate screening. Steps A to G represent the classical protocol, and the microfluidic toolbox (M1 to M5) is inserted between steps A and D. (A) Mutagenesis is induced by physical and chemical mutagens, such as UV and EMS. Mutation frequency is controlled by either the dosage used or the reaction time. (B) Prior to being selected by robots, well-separated colonies should have formed on agar plates. (C) Assuming that the automatic selection and transfer rate is 40 clones/minute (5), the process is completed in approximately 40 h for a library containing 100,000 clones. (D) One thousand 96-well microplates are required in the protocol that does not use the microfluidic toolbox. (M1 and M3) notably, if a Poisson distribution λ of 0.5 (60% empty droplets) is applied, the true rates for the generation and sorting of cell-loaded droplets are 60% lower. (M2) The conditions for incubation are the same as in steps B and M4. In this study, a 24-h incubation at 30°C is typically required for *L. lactis*. (M4) Cells in droplets are released by the addition of PFOH (1*H*,1*H*,2*H*,2*H*-perfluorooctan-1-ol). (M5) Due to the efficient enrichment (2,000-fold) using microfluidic screening, fewer than 90 clones are selected for the secondary screening in 96-well microplates.

**FIG 2:**
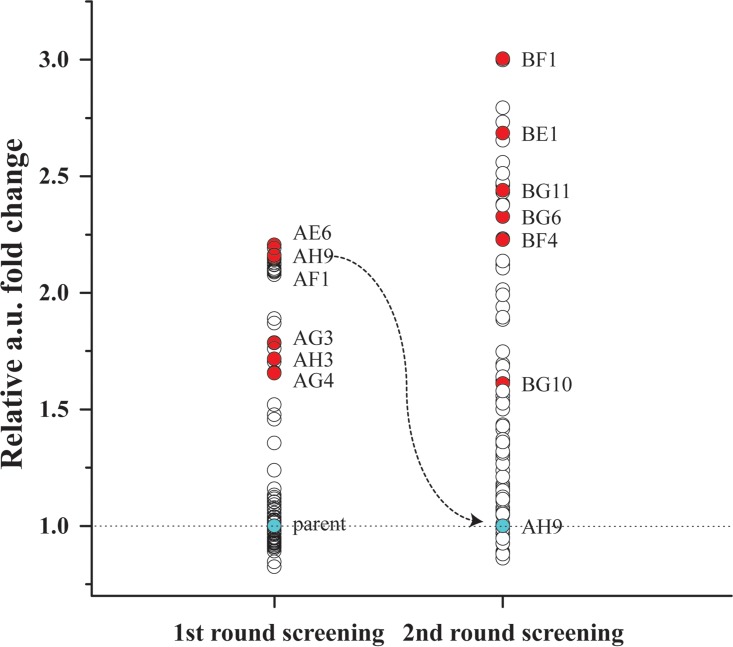
Secondary screening of riboflavin overproducers. Secondary screening of riboflavin production by the captured library was performed in 96-well plates using fluorometric assays. The parent strain JC017 and the mutant strain AH9 (blue-filled circles) were selected as the basal strains for the two rounds of mutagenesis and microfluidic screening. The relative titers of riboflavin are depicted in arbitrary units (a.u.). A few representative strains (red-filled circles) were selected for further characterization in test tubes, and their true riboflavin titers were determined using HPLC analysis (see Fig. S2 in the supplemental material).

The roseoflavin-resistant mutants, in which the FMN riboswitch has been mutated, show minor increases in the amounts of riboflavin they produce (14). We attempted to further improve the obtained mutant using random mutagenesis followed by HTS with the DBM system according to the workflow shown in Fig. 1. First, a 1-ml culture of JC017 cells (10^9^ cells) was exposed to the chemical mutagen ethyl methanesulfonate (EMS), which resulted in a library in which 1% of the original population survived (Fig. 1, step A). The surviving cells were thoroughly washed and allowed to recover in GM17 medium overnight (1:100 dilution). The library was then transferred to synthetic medium before encapsulation of single cells in aqueous droplets in oil using the microfluidics system (Fig. 1, step M1). The cell density in the aqueous phase was set to approximately 10^7^ cells/ml, resulting in approximately 0.5 cells per 50-pl-sized droplet upon encapsulation. According to the Poisson distribution, when λ = 0.5, 60% of droplets are empty, 30% of droplets contain 1 cell, and the rest contain more than 1 cell (16). Droplets were generated at approximately 1 kHz using a droplet generator (Fig. 1, step M1). Approximately 2 × 10^6^ droplets were collected in a syringe (Fig. 1, step M2). After a 24-h incubation, the droplet-containing emulsion was reinjected into the microfluidic sorting device. Mutants were screened for the presence of riboflavin based on its natural fluorescence upon exposure to blue light (excitation at 490 nm and emission at 510 nm) (Fig. 1, step M3). The sorting was performed at approximately 0.3 kHz, and a session shorter than 30 min could sort approximately 10^5^ cell-containing droplets. Approximately 50 droplets with the highest levels of fluorescence (in arbitrary units [AU]) were collected, which corresponded to a cutoff of 0.05% (see Fig. S1 in the supplemental material). The *L. lactis* cells in the collected droplets were subsequently released from the emulsions and plated on solid GM17 medium to form single colonies (Fig. 1, step M4). Characterization of 90 of the randomly picked colonies that grew in a 96-well microplate (Fig. 1, step D) revealed that one-third of the candidates produced more than 1.5-fold-higher riboflavin titers (as evaluated from the AU on the plate reader) than the parent strain, JC017 (Fig. 2). Six of the riboflavin-overproducing isolates (AH3, AG4, AE6, AF1, AG3, and AH9) were further examined in tube fermentation using riboflavin-free synthetic medium, and high-pressure liquid chromatography (HPLC) analysis revealed that all the isolates produced at least 30% more riboflavin in the supernatant than the original roseoflavin-resistant mutant, JC017 (0.82 mg/liter) (Fig. S2). The highest riboflavin titer, 2.08 mg/liter, was observed for isolate AH9 (Fig. S2). The procedure, including mutagenesis and droplet screening for improving riboflavin secretion, was repeated using AH9, and 40% of the isolates enriched in this screening round produced 1.5-fold more riboflavin than AH9 (Fig. 2). The best candidate, BE1, accumulated approximately 4 mg/liter riboflavin in the supernatant (Fig. S2), which was 4-fold higher than the amount of riboflavin secreted by JC017.

10.1128/mBio.00526-17.1 FIG S1  Distribution of fluorescence signals in the different libraries. The PMT gain was set to 0.711 and 0.665 for screening the first- and second-round mutagenesis libraries, respectively. The laser power was set at 60 mV. The dashed lines represented the cutoff position. Download FIG S1, TIF file, 0.4 MB. Copyright © 2017 Chen et al. 2017 Chen et al. This content is distributed under the terms of the Creative Commons Attribution 4.0 International license.

10.1128/mBio.00526-17.2 FIG S2  True riboflavin titers produced by the representative mutants (see Fig. 2) selected from the secondary screening. *, strains obtained from the first round of mutagenesis and screening; **, strains obtained from the second-round process. Download FIG S2, TIF file, 0.1 MB. Copyright © 2017 Chen et al. 2017 Chen et al. This content is distributed under the terms of the Creative Commons Attribution 4.0 International license.

### Whole-genome resequencing of the riboflavin overproducers AH9 and BE1.

**FIG 3:**
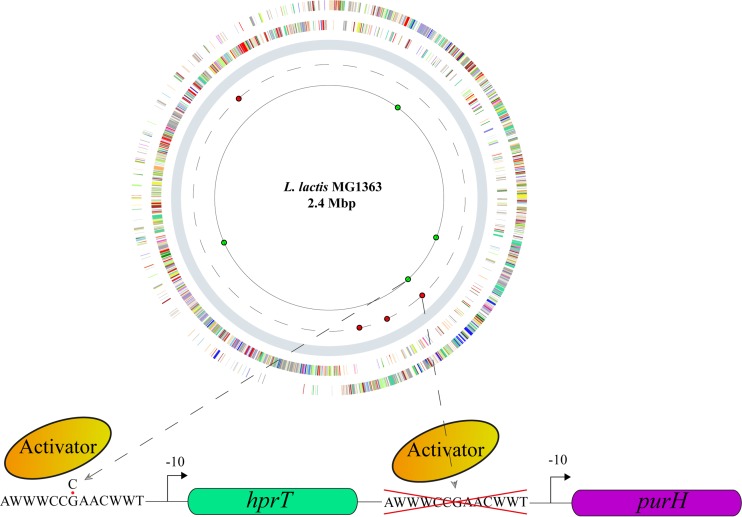
Distribution of variations in the mutants. The dashed and lined circles represent the chromosomes of the mutants AH9 and BE1, respectively. The locations of the mutations identified in AH9 are labeled with red-filled circles. The additional mutations identified in BE1 compared to AH9 are labeled with green-filled circles. A detailed list of mutations is presented in Table S1 in the supplemental material. The *purH* gene is transcribed either from its own promoter or through an operon with *hprT*.

The genomes of the two successively isolated strains AH9 and BE1 were resequenced to pinpoint the mutations responsible for the improved riboflavin production and to estimate the mutation rate. Sequencing revealed three single-nucleotide variants (SNVs) and one deletion (52-nt deleted segment, reference position 958508 to 958559 [15]) in AH9 and an additional three SNVs and one indel (insertion/deletion) in BE1 (Fig. 3; Table S1). Consistent with the known effect of the EMS mutagen (17), all SNVs were G·C-to-A·T variants (Table S1). Most mutations were located in genes with unknown functions, and none were observed in the riboflavin biosynthesis cluster *ribABGH* or its flanking regions.

10.1128/mBio.00526-17.5 TABLE S1  Variations identified in the chromosomes of isolates AH9 and BE1. Download TABLE S1, DOCX file, 0.1 MB. Copyright © 2017 Chen et al. 2017 Chen et al. This content is distributed under the terms of the Creative Commons Attribution 4.0 International license.

The intergenic region preceding the *purH* gene, which encodes a 5-aminoimidazole-4-carboxamide ribonucleotide (AICAR) transformylase that catalyzes the second step in purine *de novo* synthesis, had been deleted in AH9 (Fig. 3). The missing segment contains a PurBox regulatory sequence, which is known to be involved in the transcriptional activation of *purH* (18). In BE1, a mutation (G-to-A transition, reference position 957859 [15]) was identified in another PurBox regulatory sequence preceding the *hprT* gene, which also forms an operon with *purH* (Fig. 3) (18).

The wild-type and mutated regions upstream from *purH* and *hprT-purH* were fused to the reporter gene *gusA*, which encodes β-glucuronidase, and were integrated into the chromosome of the wild-type background (*L. lactis* MG1363) to determine the effects of the two mutations on expression. The results of a β-glucuronidase assay indicated that the mutations resulted in reduced expression. The mutation preceding *hprT* resulted in 3-fold-lower expression, and the deletion of the second PurBox reduced expression from the promoter upstream from *purH* to undetectable levels (Fig. S3).

10.1128/mBio.00526-17.3 FIG S3  Expression of genes related to purine and riboflavin synthesis in both the parent and mutant strains. + purine, 200 mg/liter guanosine was added. The data are average results from two independent replications. ND, not detectable. Download FIG S3, TIF file, 0.3 MB. Copyright © 2017 Chen et al. 2017 Chen et al. This content is distributed under the terms of the Creative Commons Attribution 4.0 International license.

### Identification of triggers for riboflavin overproduction.

**FIG 4:**
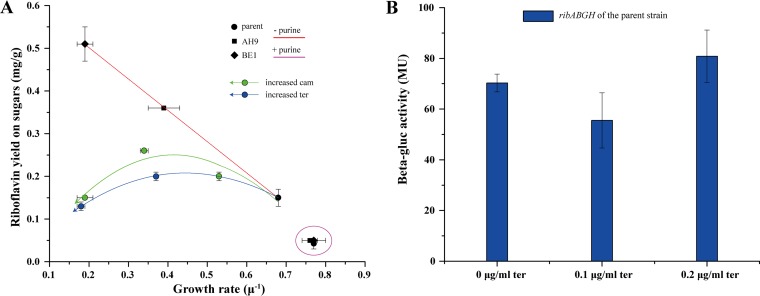
Effects of purine metabolism and growth rate on riboflavin production. (A) Effects of purine metabolism and growth rate on riboflavin yield. Ter, tetracycline; Cam, chloramphenicol. (B) Effect of inhibiting anabolism on the expression of the riboflavin synthesis cluster in the parent strain JC017. The data are the average results and standard deviations from two independent experiments. Error bars show standard deviations.

Purine *de novo* biosynthesis provides the precursor GTP for riboflavin biosynthesis, and more importantly, purine nucleotides are essential for energy metabolism and various anabolic processes, such as DNA, RNA, and protein synthesis. We observed a link between riboflavin production and growth rates for the different mutants, as the specific growth rate decreased while riboflavin production increased (Fig. 4A). Since the expression of purine biosynthesis genes was reduced in AH9 and BE1, we decided to test whether supplementation with purines (200 mg/liter guanosine) would influence the growth and riboflavin secretion of the original roseoflavin-resistant strain JC017 and the DBM isolates AH9 and BE1. Supplementation indeed resulted in fast growth and similar growth rates for all three strains, and riboflavin production by the isolates AH9 and BE1 decreased to levels comparable to that of the parent strain JC017 (Fig. 4A).

As a simple test to determine whether riboflavin overproduction was solely a consequence of slow growth or whether other factors were involved, we decided to reduce the growth rate of the parent strain JC017 by adding low concentrations of chloramphenicol or tetracycline, an approach that has commonly been used to study growth-related effects (19). However, compared to the effects of the mutations, reducing the growth rate by adding antibiotics had only a moderate effect on riboflavin overproduction in JC017 (Fig. 4A).

**FIG 5:**
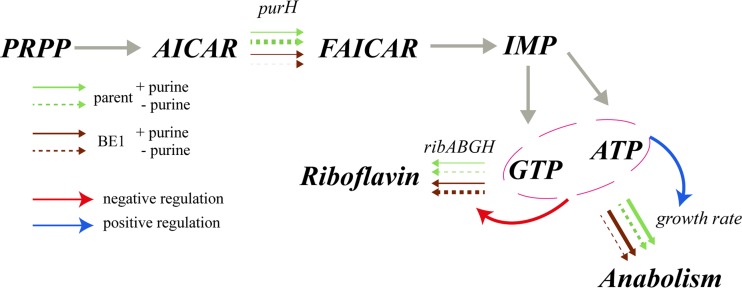
Pleiotropic effects of the *purH*-related mutations on riboflavin synthesis and anabolism. The line widths of the arrows (green and brown) represent the relative expression levels of *purH* and *ribABGH*. Differences in anabolism are indicated by the relative growth rates. The data presented for *purH* were summed from the results for *purH* and the *hprT-purH* fusion. Detailed expression data are presented in Fig. S3 in the supplemental material. + purine, 200 mg/liter guanosine was added; PRPP, phosphoribosyl pyrophosphate; AICAR, 5-aminoimidazole-4-carboxamide ribonucleotide; FAICAR, 5-formamidoimidazole-4-carboxamide ribotide.

We then determined the expression of the riboflavin biosynthesis cluster *ribABGH* in both the parent strain JC017 and the mutant strain BE1 by fusing its promoter with *gusA*. The β-glucuronidase activities indicated that *ribABGH* expression was increased 6-fold in BE1 compared with that in JC017, and the addition of guanosine (200 mg/liter) restored the *ribABGH* expression in BE1 to the levels observed in JC017 (Fig. 5; Fig. S3). The antibiotic-induced reduction in the growth rate did not alter the *ribABGH* expression in JC017 (Fig. 4B), although a slight increase in the riboflavin titer was observed.

### Optimization of riboflavin production during fermentation and riboflavin biofortification in milk.

Although purine starvation induced riboflavin overproduction in the mutants, the starvation also significantly inhibited growth, which, in principle, has a negative effect on volumetric productivity and strain stability. We decided to add small amounts of guanosine to the fermentation broth to achieve a two-stage fermentation, where rapid growth is observed in the first phase to ensure the rapid accumulation of biomass, followed by a production phase characterized by slow growth due to guanosine depletion. The approach was successful (Fig. S4), and the DBM isolate BE1 generated 6.5 mg/liter riboflavin, with a higher volumetric productivity than in the fermentation without the initial purine addition.

10.1128/mBio.00526-17.4 FIG S4  Batch fermentation of riboflavin by various strains. The data are average results from two independent batches of fermentation. Download FIG S4, TIF file, 0.1 MB. Copyright © 2017 Chen et al. 2017 Chen et al. This content is distributed under the terms of the Creative Commons Attribution 4.0 International license.

**FIG 6:**
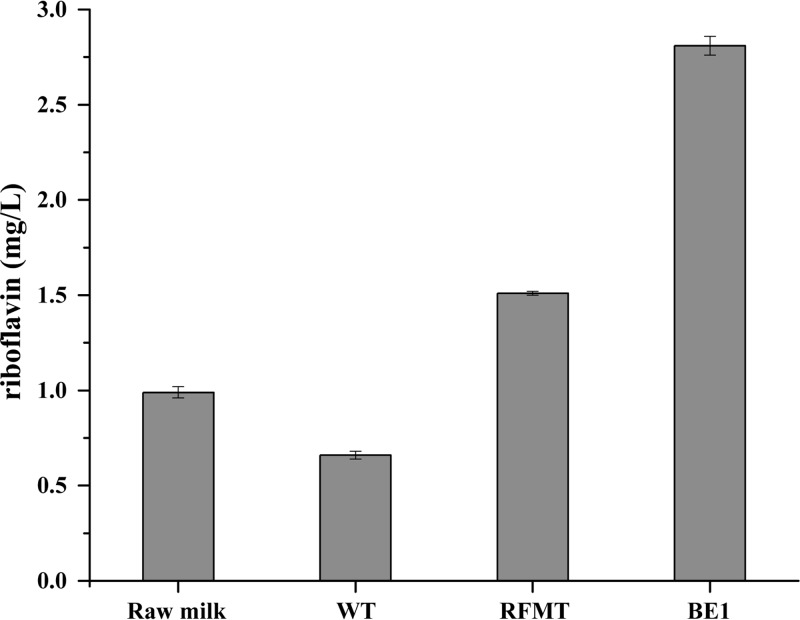
Riboflavin content of milk after fermentation by different strains. WT, *L. lactis* MG1363; RFMT, the parent strain JC017 derived from MG1363 by roseoflavin selection. The data are the averages and standard deviations from three biologically independent replications. Error bars show standard deviations.

The mutants were isolated from a synthetic medium with a composition that is quite different from that of milk. Due to their non-GMO nature (2, 12), the isolated mutants could be used to produce fermented foods fortified with riboflavin. Therefore, we decided to test their performance in milk. Single colonies of the wild-type (*L. lactis* MG1363), the parent (the original roseoflavin-resistant mutant derived from *L. lactis* MG1363, JC017) and the BE1 strains were inoculated into 50 ml of milk. After a 24-h incubation, all growth had ceased due to acidification of the milk, and the riboflavin content was determined. The wild-type strain reduced the natural riboflavin content of the milk from 0.99 mg/liter to 0.66 mg/liter (Fig. 6); however, both the parent strain and the BE1 strain increased the riboflavin content, to 1.51 mg/liter and 2.81 mg/liter, respectively.

## DISCUSSION

With a lack of prior knowledge regarding how to alter a phenotypic property of microorganisms, classic random mutagenesis followed by screening for mutants of interest has been extensively used for this purpose, and in combination with reverse engineering, the underlying determinants could be clarified (4). In contrast, metabolic engineering is a more targeted approach that has been used extensively to optimize cell factories when prior knowledge of metabolic pathways is available; however, the genes that need to be mutated are not always obvious (20). In our study, the objective was to improve riboflavin production; thus, one would assume that a successful strategy would involve strengthening the metabolic flux toward GTP, which is an essential riboflavin precursor (21). Very unexpectedly, the mutants generated by mutagenesis showed the opposite result, as impairment in purine biosynthesis in the mutants triggered the riboflavin overproduction (Fig. 3 and 4).

Purine biosynthesis provides the vital building blocks required for the synthesis of DNA, RNA, the energy carriers involved in metabolism (ATP and GTP), and molecules involved in cellular signaling, such as cyclic AMP (cAMP), cGMP, and ppGpp (22). When the cultures were not supplemented with purines, the mutants grew more slowly due to the mutations in the genes involved in purine metabolism (Fig. 4A). Thus, we artificially inhibited the growth of the parent strain JC017 by adding antibiotics targeting protein synthesis to test whether slow growth explained the increased riboflavin production. Indeed, the riboflavin production was increased in JC017, but only slightly. Slow growth has been shown to promote the production of vitamins derived from GTP, and this effect is due to a surplus of GTP from the reduced anabolism for vitamin biosynthesis under these circumstances (19). In our case, the slow growth of the mutants was a direct consequence of purine starvation (Fig. 4A and 5), indicating that other factors in addition to a slow anabolism are involved. In the microorganisms that naturally produce riboflavin, such as some fungi, the expression of riboflavin biosynthesis genes is normally induced during the transition from the vegetative phase to the stationary phase, and here, the growth rate is the trigger (23). However, when we inhibited the growth of the original roseoflavin-resistant mutant JC017 by adding tetracycline, the expression of the riboflavin operon was not affected (Fig. 4B).

For the mutant BE1, the expression of the riboflavin operon was increased 6-fold compared to its expression in JC017, but only in the absence of purine nucleosides (Fig. 5; see also Fig. S3 in the supplemental material). In bacteria, riboflavin biosynthesis is normally tightly controlled by an FMN riboswitch. This riboswitch had been eliminated in the original roseoflavin-resistant mutant JC017, but the repressive effect of guanosine indicates that other regulatory mechanisms are active. Riboflavin production in *Ashbya gossypii* is activated by sporulation, which is negatively regulated by purine derivatives, such as cAMP (24). Further investigation is required to determine whether the effects observed in the *L. lactis* mutants are mediated by cAMP or other purine derivatives.

In a random mutagenesis experiment, it is difficult to achieve a sufficiently high mutation rate to facilitate selection of the relevant phenotype, since the chances of introducing other unwanted mutations are also concomitantly increased (25). Unwanted mutations may have deleterious effects on bacterial fitness, and Maisnier-Patin et al. have estimated that the presence of 50 nontarget mutations per genome in bacteria results in an approximately 40% average decrease in the growth rate, whereas the presence of 5 nontarget mutations reduced this value to 10% (26). According to a study by Kibota and Lynch, 10% of spontaneous mutations arising during evolution have a negative effect on bacterial fitness (27). This effect may be exacerbated in an industrial setting, where microorganisms must cope with large fluctuations in nutrient availability and temperature and are exposed to various stresses (28). Moreover, certain fitness-irrelevant metabolic traits, such as the ability to form certain fermentation products, e.g., acids, flavors, and compounds contributing to texture, must be considered when evaluating microorganisms applied in food fermentation (29). Thus, the number of unwanted mutations is preferred to be minimized.

Often, only a few of the mutations introduced by random mutagenesis are actually linked to the desired property, and unwanted mutations constitute the major fraction of the identified mutations, particularly when a high dose of mutagen has been applied to facilitate selection (10, 20). Therefore, the use of a milder mutagenesis approach is preferred, and researchers can compensate for the reduced frequency with efficient screening approaches. In the current study, we used a DBM system to achieve this goal. The mutants obtained using the DBM system were verified using the low-throughput equipment (96-well plates with plate reader), which was manageable due to the efficient primary enrichment using droplet sorting, which resulted in a large fraction (30 to 40%) of true riboflavin overproducers (Fig. 2), which were further verified using HPLC analysis (Fig. S2). Moreover, the stable mutation rate (Fig. 3) and high fraction of true riboflavin overproducers that resulted from droplet sorting (Fig. 2) in the iterative rounds of the mutagenesis screen showed that the entire procedure was easily controllable and highly efficient. In this study, the total operating time required for one round of droplet generation and sorting was less than 1 h (Fig. 1, steps M1 and M3). Using an automated liquid handling system for the same task on 96-well microplates would require more than one day for colony selection and more substantial loads to read the plates (Fig. 1, steps C and D). Although additional incubation steps are required between droplet generation and sorting when using the DBM system (Fig. 1, step M2), the efficient enrichment (2,000 times) with DBM screening also significantly reduced the expenditures for consumables. Using this paradigm, one thousand 96-well microplates and more consumables for colony picking and culturing (6) would be used for the same project if the classical strain development procedure was followed (Fig. 1).

The efficiency of the DBM screening system allowed us to easily capture mutants with the desired phenotype, and the low mutation rate allowed us to easily link the mutation to the phenotype. In principle, the approach using mild random mutagenesis in combination with DBM screening can be applied to all types of cells. In particular, this approach would be useful for prokaryotes, which generally possess smaller genomes, as genome sequencing and reverse engineering are quite simple, thus avoiding complicated systematic approaches (10, 20). In this study, we took advantage of the natural fluorescence of riboflavin for detection, but other approaches can easily be used as well. The recent development of GFP-based biosensor technology has been successfully applied to isolate microorganisms producing riboflavin and other compounds (9, 30).

Compartmentalization in water-in-oil droplets sequesters individual bacterial cells in an emulsion bulk, and thus, the performance of individual cells in producing chemicals and proteins is traceable. Although FACS-based screening can be performed a thousand times faster than DBM screening, the lack of compartmentalization does not provide FACS with the capacity to perform the same tasks (31). Recently, the emergence of double-layer droplet technology (a water-in-oil-in-water emulsion) has allowed researchers to accommodate the sorting of droplets on a commercial FACS machine, which has been shown to be 10 times faster than screening on a microfluidics-based sorter (8, 32). Nevertheless, there are still several limitations to sorting double-layer droplets in FACS for the purpose of screening. First, the small size of the nozzle used in a common FACS setup does not readily accommodate the large droplets that are normally used to propagate microbes and accumulate extracellular metabolites (7, 8, 11). The size of double-layer droplets is reduced by a hypertonic solution as they pass through the nozzle, but the concomitant osmotic stress will have detrimental effects on bacterial fitness, potentially affecting further recovery (8). Second, further modifications of contents, such as droplet merging after encapsulation, are restricted in double-layer droplets (31), which may be important if cell growth and analysis are performed in a two-step manner (7).

In summary, DBM is a powerful tool for isolating useful riboflavin-producing *L. lactis* variants. In principle, the tool can be used on any cell type to examine other properties, and DBM is perfectly suited for optimizing industrially used cell factories or microorganisms used for food fermentation due to its highly efficient sorting process. Based on our results showing that large populations can be screened rapidly, harsh mutagenesis is no longer required to obtain a desired phenotype.

## MATERIALS AND METHODS

### Bacterial strains and culture medium.

The *L. lactis* strains used in this study are listed in Table S2 in the supplemental material. M17 broth (Sigma-Aldrich, St. Louis, MO, USA) supplemented with 0.5% glucose (GM17 broth) was used for the general cultivation of the *L. lactis* strain. Nucleoside- and riboflavin-free SA medium supplemented with 0.5% glucose and 15 mM acetate instead of lipoic acid was used for the isolation and assessment of riboflavin overproducers (33). When necessary, guanosine was supplied to the SA medium at a concentration of 5 mg/liter or 200 mg/liter. The *Escherichia coli* strains used for cloning were grown aerobically in Luria-Bertani (LB) broth (34). Different antibiotics were used to maintain the plasmids in the strains. Erythromycin concentrations of 150 µg/ml and 5 µg/ml were used for the *E. coli* and *L. lactis* strains, respectively. A chloramphenicol concentration of 5 µg/ml was used for *L. lactis*. For testing riboflavin fortification in milk, the *L. lactis* strains derived from *L. lactis* MG1363 were transformed with plasmid pLB712 prior to milk fermentation (35). Ultrahigh-temperature-processed (UHT) whole milk (Arla Food A.m.b.a, Viby J, Denmark) was used for testing riboflavin production in milk by different strains.

10.1128/mBio.00526-17.6 TABLE S2  List of *L. lactis* strains used in this study. Download TABLE S2, DOCX file, 0.04 MB. Copyright © 2017 Chen et al. 2017 Chen et al. This content is distributed under the terms of the Creative Commons Attribution 4.0 International license.

### Isolation of the roseoflavin-resistant mutant.

A previously described procedure (14) was used to isolate the roseoflavin-resistant mutant on riboflavin-free SA agar plates containing 100 mg/liter roseoflavin (MP Biomedicals, Santa Ana, CA, USA).

### Fabrication of microfluidic devices.

The two chips used for the encapsulation and sorting of the droplets are both composed of a structured polydimethylsiloxane (PDMS) slab bonded to a glass slide. A 10:1 (wt/wt) mixture of PDMS (Sylgard 184; Dow Corning, Auburn, MI, USA) was cast over a mold created using regular UV photolithography to pattern a layer of SU-8 2075 epoxy resist (MicroChem, Westborough, MA, USA) on a 4-inch silicon wafer. The PDMS was cured overnight at 60°C, cut into separate devices, and carefully removed from the mold. Holes for inlets and outlets were created using biopsy punches (Ø:0.75 mm). Both the glass slides and PDMS parts were exposed to 50-W, 13.56-MHz air plasma for 60 s (Atto plasma cleaner; Diener, Ebhausen, Germany) and then immediately bonded, placed under a weight (400 g/slide), and incubated at 90°C for 10 min. After cooling, the channels were flushed with filtered Aquapel and subsequently quickly purged by applying a vacuum to the outlets. The sorting chips were heated on an 85°C hotplate, allowing a low-temperature solder wire (Indalloy number 19; Indium Corp., Clinton, NY, USA) to be inserted and fill the electrode cavities. Before cooling the device, small pieces of wire were inserted as connectors to the electrodes.

### Experimental setup of microfluidic devices.

The microfluidic setup was inspired by the system described by Mazutis et al. (16). The sorting chip was placed on a custom-built fluorescence microscope, where a 488-nm laser (06-01; Cobolt, Solna, Sweden) was guided through a 1,000-mm cylindrical lens (LJ1516RM-A; Thorlabs, Newton, NJ, USA) and a 10× microscope objective (N10X-PF; Thorlabs) to form a narrow line for fluorescence detection. The fluorescent light emitted was transmitted through a dichroic mirror (MD499; Thorlabs) and a bandpass filter (MF530-43; Thorlabs) before being measured by a photomultiplier tube (PMT) (PMTSS; Thorlabs). A field-programmable gate array (FPGA) (PCIe-7842R; National Instruments, Austin, USA) was programmed to sample the PMT signal every 7 µs (>143 kHz) and continuously evaluate both the signal width and intensity. When a signal fell within the preset gating, the on-chip electrodes were activated with a square voltage wave (15 kHz) amplified to ±400 V (Trek 623B; Trek, Inc., Lockport, NY, USA). The fluidic inlets were connected to a vertically oriented syringe pump (PHD 2000; Harvard Apparatus, Inc., Holliston, MA, USA) using PTFE tubing (TW30; Adtech, Stroud, United Kingdom) with an internal diameter of 320 µm. Fluorinated ethylene propylene (FEP) tubing (JR-T-6794-M10; VICI-Jour, Schekon, Switzerland) with an inner diameter of 100 µm was used for the fluidic outlets to ensure that the sorted drops were immediately pushed out of the tubing and collected in Eppendorf tubes.

### Chemical mutagenesis.

*L. lactis* strains were grown in GM17 broth overnight at 30°C to achieve a density of 10^9^ cells/ml. Mutagenesis was conducted by adding 3% ethyl methanesulfonate (EMS; Sigma, St. Louis, MO, USA) to the overnight culture, followed by a 3-h incubation at 30°C. Samples were then washed twice with fresh GM17 to remove the residual EMS. Afterward, samples were used for further experiments or frozen in 20% glycerol at −80°C. For determining the survival rate, the EMS-treated/untreated samples were plated on GM17 agar plates with appropriate dilutions. After a 48-h incubation at 30°C, the colonies were counted to estimate the survival rate.

### Encapsulation in droplets.

The library was first diluted into SA medium at a density of 10^7^ cells/ml. Both the aqueous (culture) and oil (Dolomite Pico-Surf 2, 5%; Royston, United Kingdom) inflow rates were set to 10 µl/min, which normally resulted in a droplet size of 50 pl. A 1-ml syringe (BD Medical, Sandy, UT, USA) was connected to the outlet to collect the emulsions. After incubation, the fraction of cell-loaded droplets was estimated by examining the monodisperse emulsions on a microscope.

### Fluorescence-based sorting.

After incubation, samples in syringes were reinjected into the sorting chip at a rate of 1 µl/min, and the continuous phase of the Novec 7500 (3M, St. Paul, MN, USA) was set to 10 µl/min to allow sufficient space between two adjacent droplets. The fluorescence intensity of each drop was measured and compared to a preset gating level. For each sorting operation, the electrical field was triggered for 1,750 µs after a 250-µs delay to allow droplets to pass the gap between the laser spot and the electrodes. Samples were collected directly from the outlet channels into a 1.5-ml Eppendorf tube containing 300 µl of phosphate-buffered saline (PBS) buffer (pH 7.4).

### Release of cells from droplets.

Samples obtained from sorting were first centrifuged at 100 × *g* for 30 s to ensure that the emulsions were floating in the layer between the oil and aqueous phases. After removing as much of the bottom layer (oil phase) as possible by pipetting, 300 µl of PFOH (1*H*,1*H*,2*H*,2*H*-perfluorooctan-1-ol) (Sigma, St. Louis, MO, USA) was added to disrupt the droplets and release the cells into the aqueous layer. The aqueous phase containing the cells was spread directly onto GM17 agar plates at an appropriate dilution and incubated.

### Genome resequencing and mutation identification.

Genomic DNA was purified from the mutant using a DNeasy blood and tissue kit (Qiagen, Hilden, Germany), and the quality was assessed using DNA electrophoresis and a NanoDrop 1000 spectrophotometer (Thermo Fisher Scientific, Waltham, MA, USA). Genome sequencing was performed by Macrogen (Seoul, South Korea). Briefly, 2 µg of genomic DNA was randomly sheared using a nebulizer (Illumina, San Diego, CA, USA), and the ends were repaired using polynucleotide kinase (Illumina) and Klenow enzyme (Illumina). The 5′ ends of the DNA fragments were phosphorylated, and a single adenine base was added to the 3′ ends using Klenow exo+ (Illumina). Following the ligation of a pair of Illumina adaptors to the repaired ends, the DNA was amplified in 10 cycles using adaptor primers (Illumina), and fragments of approximately 150 bp were isolated using agarose gel electrophoresis. Sequencing libraries were quantified with a 2100 BioAnalyzer DNA 1000 chip (Agilent, Santa Clara, CA, USA), as well as the PicoGreen fluorescence assay (Invitrogen, Carlsbad, CA, USA). Clusters were generated on an Illumina cluster station (Illumina) using 11 pmol of sequencing libraries. Thirty-eight sequencing cycles were performed using the Illumina Genome Analyzer IIx system (Illumina) according to the manufacturer’s specifications. CLC Genomics Workbench (Qiagen, Hilden, Germany) was used to map the reads, detect single-nucleotide variations (SNVs) and insertion/deletion events (INDELs), and identify genomic rearrangements using the published genomic sequence of *L. lactis* MG1363 as the reference (15).

### Quantification of riboflavin secretion.

The extracellular riboflavin concentration was determined using reversed-phase HPLC. A µBondapak C_18_ column (Waters Associates, Milford, USA) was used, and the mobile phase consisted of water-methanol-acetic acid (68:32:0.1 [vol/vol]). Detection was performed using UV spectroscopy at 270 nm. Samples were pretreated using a previously described acetic acid extraction procedure prior to analysis of the riboflavin content in milk (36).

### β-Glucuronidase activity assay.

The fragments containing promoters and 5′ untranslated regions, including the Shine-Dalgarno sequence, were PCR amplified using the primers listed in Table S3 to construct the strains for the β-glucuronidase assay. These PCR fragments were cloned into the XbaI site of the pLB85 vector (37) using Gibson assembly (38). The constructs were integrated into the *attB* site in the chromosome of the *L. lactis* strain bearing the pLB65 vector that expresses the TP901-1 integrase (37).

10.1128/mBio.00526-17.7 TABLE S3  List of primers used in this study. Download TABLE S3, DOCX file, 0.05 MB. Copyright © 2017 Chen et al. 2017 Chen et al. This content is distributed under the terms of the Creative Commons Attribution 4.0 International license.

The activity of the β-glucuronidase enzyme was assessed using *para*-nitro-β-glucuronic acid (PNPG) (Sigma, St. Louis, MO, USA) as the substrate according to a previously described procedure (39).
