# Supplementary figures and images for: Finding the Needle in the Haystack—the Use of Microfluidic Droplet Technology to Identify Vitamin-Secreting Lactic Acid Bacteria
Source: mBio. 2017 May 30;8(3):e00526-17. doi: 10.1128/mBio.00526-17 (PMC5449655; doi:10.1128/mBio.00526-17)

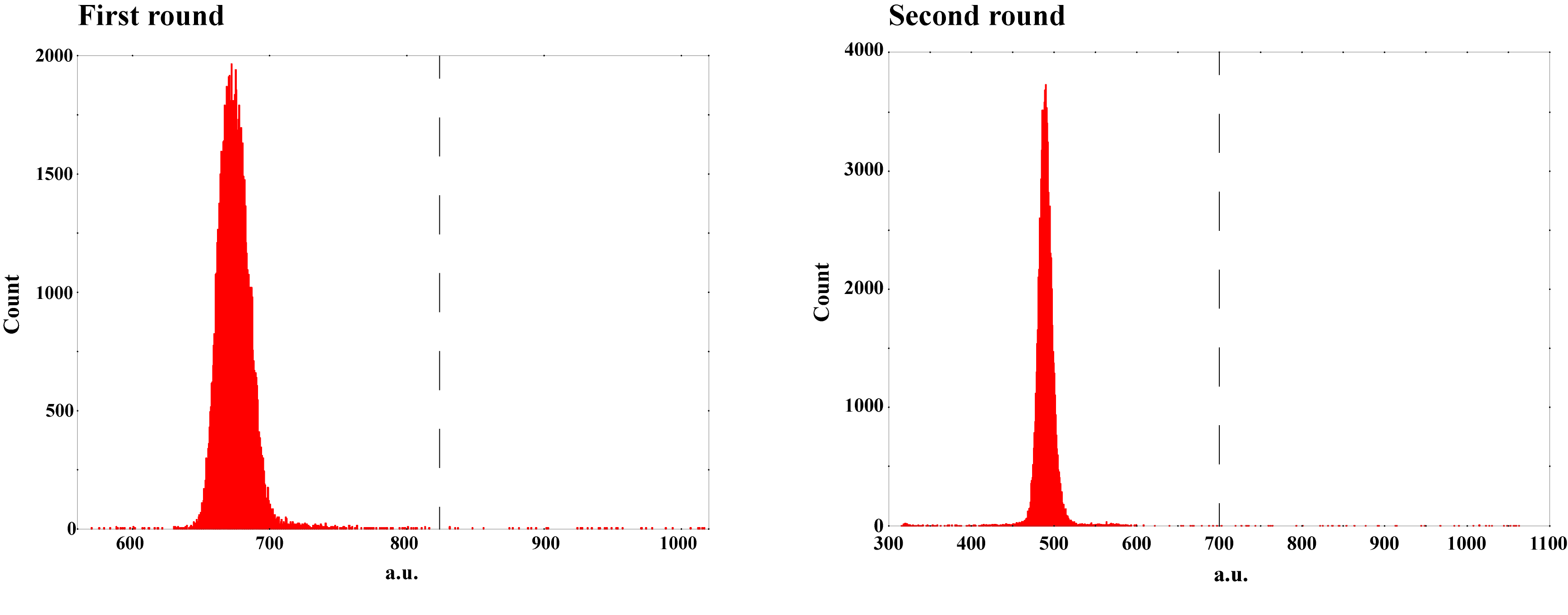

Supplement: FIG S1 [file mbo003173322sf1.tif]

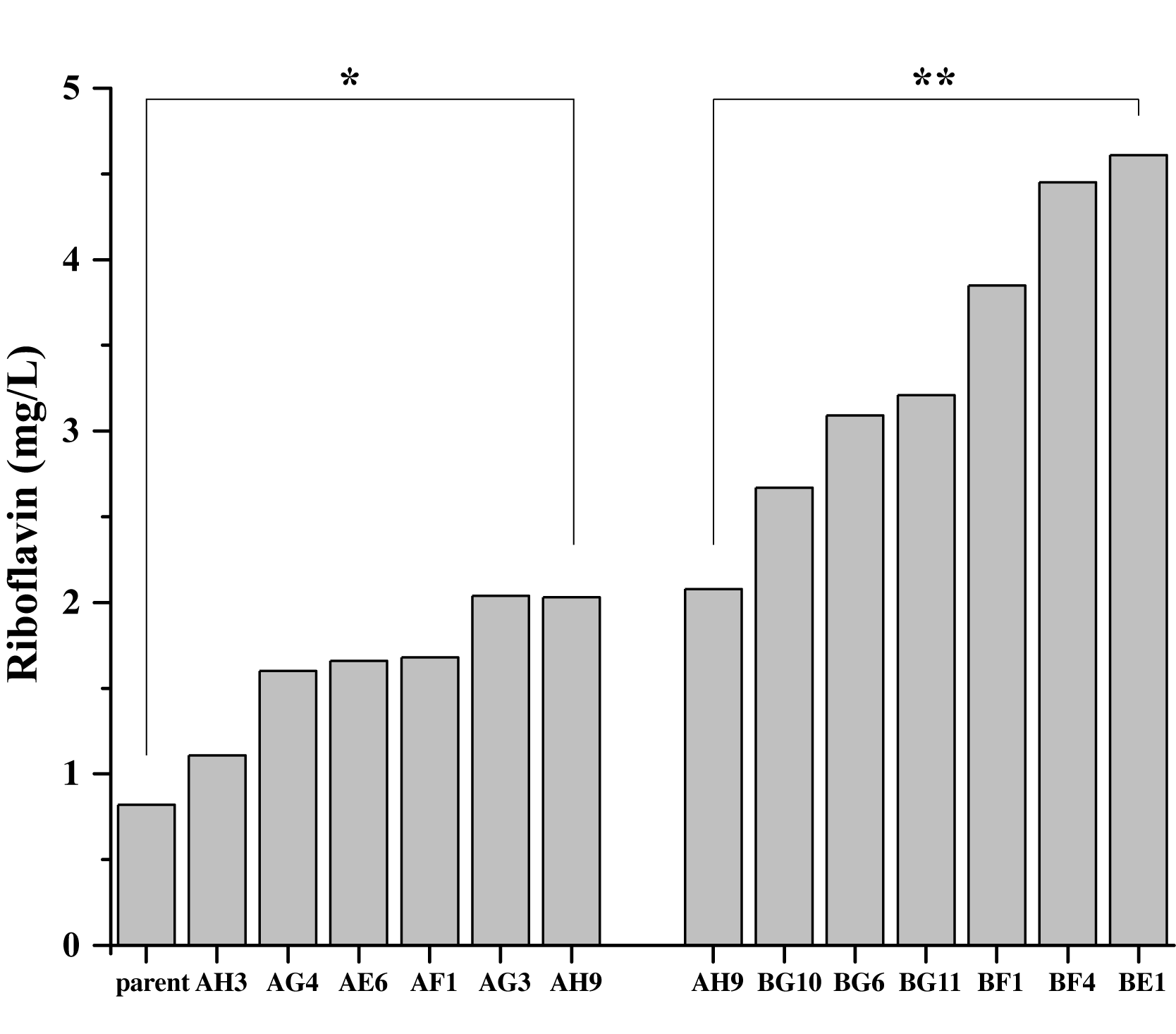

Supplement: FIG S2 [file mbo003173322sf2.tif]

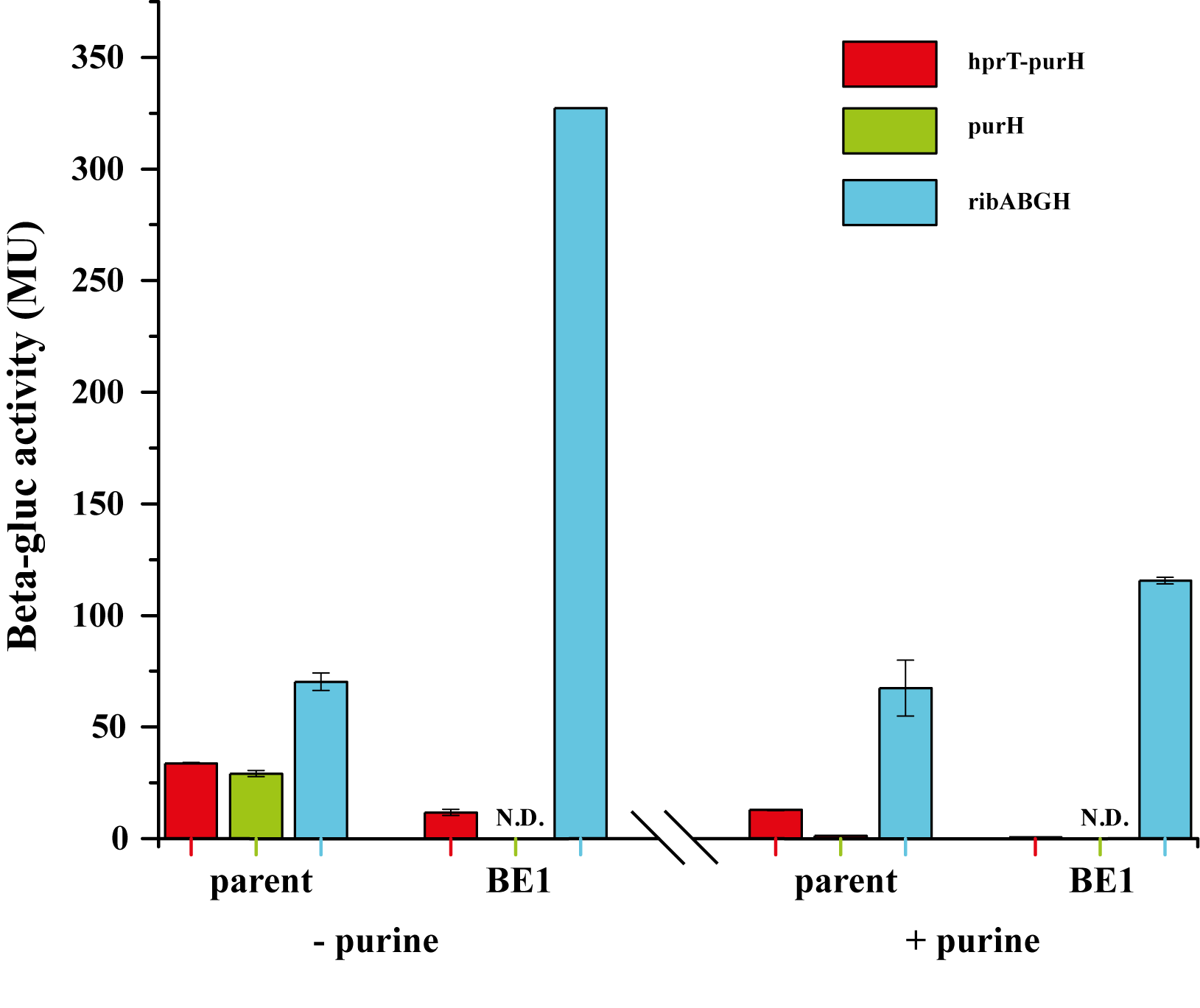

Supplement: FIG S3 [file mbo003173322sf3.tif]

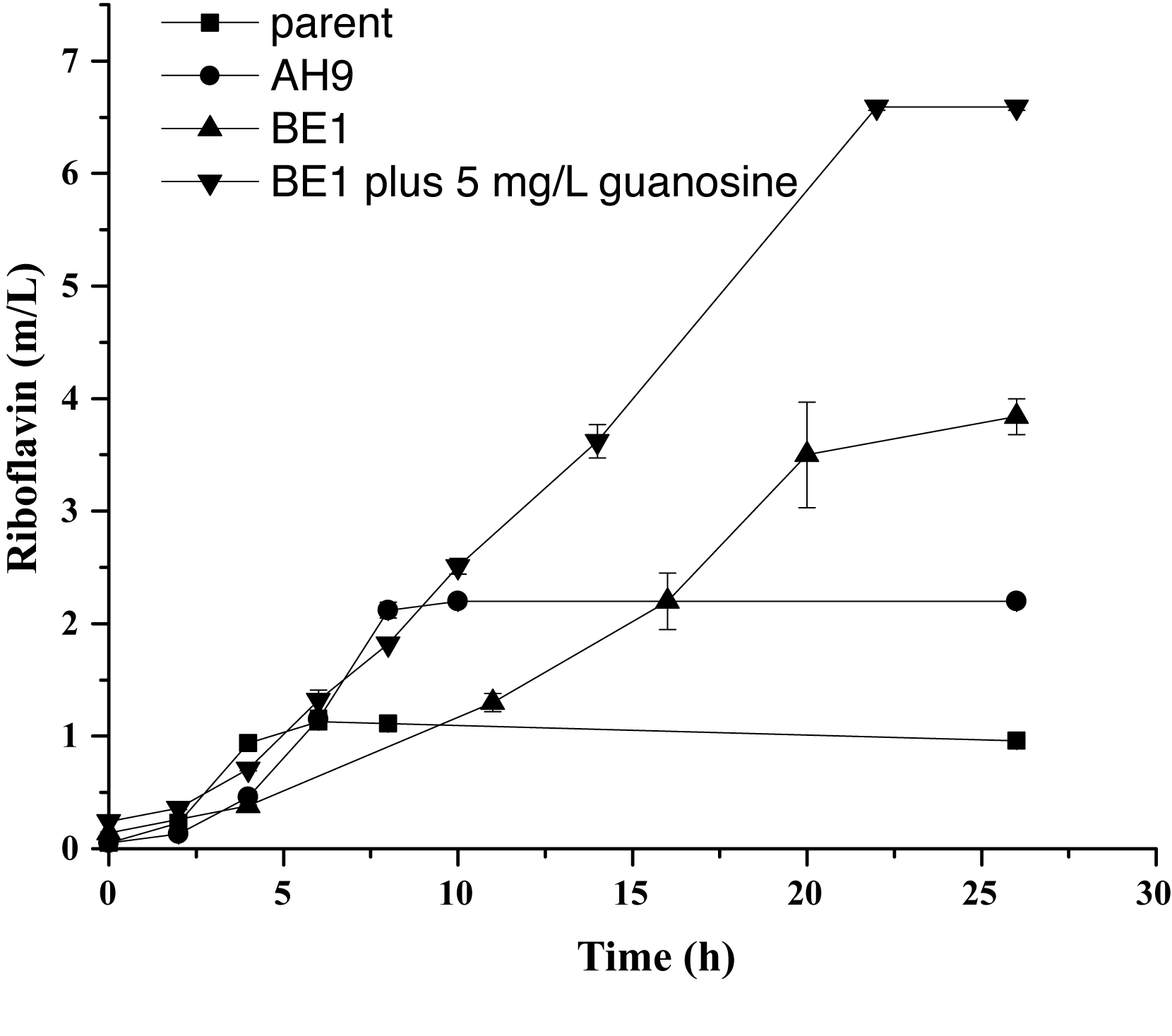

Supplement: FIG S4 [file mbo003173322sf4.tif]
