# Supplementary material for: Finding the Needle in the Haystack—the Use of Microfluidic Droplet Technology to Identify Vitamin-Secreting Lactic Acid Bacteria
Source: mBio. 2017 May 30;8(3):e00526-17. doi: 10.1128/mBio.00526-17 (PMC5449655; doi:10.1128/mBio.00526-17)
Supplement: TABLE S1 [file mbo003173322st1.docx]

Table S1. Variations identified on the chromosomes of the mutant strains AH9 and BE1

| Strain name | |  |  |  |  |  |  |
| --- | --- | --- | --- | --- | --- | --- | --- |
| AH9 | BE1 | Reference   position | Variation  type | Changed  nucleotide | Gene | Protein | Amino acid  change |
| ● | ■ | 958508 - 958509 | DEL |  |  |  |  |
| ● | ■ | 1085831 | SNV | C → T | *llmg_1124* | Hyprothetical protein | Gly → Arg |
| ● | ■ | 1173394 | SNV | C → A | *llmg_1205* | Hyprothetical protein | Slient mutation |
| ● | ■ | 2231931 | SNV | G → A | *llmg_2272* | Hyprothetical protein | Ser → Leu |
|  | ■ | 262369 | InDel | T → - | *Intergenic* |  |  |
|  | ■ | 778865 | SNV | G → A | *Intergenic* |  |  |
|  | ■ | 957859 | SNV | G → A | *Intergenic* |  |  |
|  | ■ | 1730863 | SNV | C → T | *llmg_1748* | Hyprothetical protein | Slient mutation |

DEL: deletion.
