# Supplementary material for: Finding the Needle in the Haystack—the Use of Microfluidic Droplet Technology to Identify Vitamin-Secreting Lactic Acid Bacteria
Source: mBio. 2017 May 30;8(3):e00526-17. doi: 10.1128/mBio.00526-17 (PMC5449655; doi:10.1128/mBio.00526-17)
Supplement: TABLE S2 [file mbo003173322st2.docx]

| **Table S2.** List of *L. lactis* strains used in this study. | | |  |
| --- | --- | --- | --- |
| *L. Lactis* strains | Characteristics | Reference or source |  |
| *L. lactis* MG1363 | A prophage-cured and plasmid-free derivative of *L. lactis* subsp. *cremoris* NCDO 712 | (35) |  |
| JC017 | A roseoflavin-resistant mutant derived from *L. lactis* MG1363 | This study |  |
| AH9 | A riboflavin overproducer isolated from the EMS mutagenized library of JC017 using droplet sorting | This study |  |
| BE1 | A riboflavin overproducer isolated from the EMS mutagenized library of AH9 using droplet sorting | This study |  |
