# Supplementary material for: Finding the Needle in the Haystack—the Use of Microfluidic Droplet Technology to Identify Vitamin-Secreting Lactic Acid Bacteria
Source: mBio. 2017 May 30;8(3):e00526-17. doi: 10.1128/mBio.00526-17 (PMC5449655; doi:10.1128/mBio.00526-17)
Supplement: TABLE S3 [file mbo003173322st3.docx]

Table S3. List of primers used in this study

| Name | Sequence |
| --- | --- |
| hprT_P_fwd | ATCTCATGCATAGAAGGAATCCATATGACTAGTAGATCCTTTAGATTTGGCTGTGATTA |
| hprT_P_rev | TAGCTAGCTAGCGGCCGCGAATTCTGCAGGTCGACTC TAGTTCTCTTGTAAATAAGACTTC |
| purH_P_fwd | ATCTCATGCATAGAAGGAATCCATATGACTAGTAGAT CCTAAGAATTTTTGATTAAAGAG |
| purH_P_rev | TAGCTAGCTAGCGGCCGCGAATTCTGCAGGTCGACTC TAGTTCTTTGTCTGAAACACT |
| rib_P_fwd | ATCTCATGCATAGAAGGAATCCATATGACTAGTAGAT CCTCATTTATTTTTATTTCTTGC |
| rib_P_rev | TAGCTAGCTAGCGGCCGCGAATTCTGCAGGTCGACTC TAGTAAATTCATAAAATGCTCAT |
